# A Bioengineered Three-Dimensional Cell Culture Platform Integrated with Microfluidics To Address Antimicrobial Resistance in Tuberculosis

**DOI:** 10.1128/mBio.02073-16

**Published:** 2017-02-07

**Authors:** Magdalena K. Bielecka, Liku B. Tezera, Robert Zmijan, Francis Drobniewski, Xunli Zhang, Suwan Jayasinghe, Paul Elkington

**Affiliations:** a NIHR Respiratory Biomedical Research Unit, Clinical and Experimental Sciences Academic Unit, Faculty of Medicine, University of Southampton, Southampton, United Kingdom; b Faculty of Engineering, University of Southampton, Southampton, United Kingdom; c Department of Infectious Disease, Imperial College London, London, United Kingdom; d BioPhysics Group, UCL Institute of Biomedical Engineering, UCL Centre for Stem Cells and Regenerative Medicine and UCL Department of Mechanical Engineering, University College London, London, United Kingdom; e Institute for Life Sciences, University of Southampton, Southampton, United Kingdom; Sequella, Inc.

## Abstract

Antimicrobial resistance presents one of the most significant threats to human health, with the emergence of totally drug-resistant organisms. We have combined bioengineering, genetically modified bacteria, longitudinal readouts, and fluidics to develop a transformative platform to address the drug development bottleneck, utilizing *Mycobacterium tuberculosis* as the model organism. We generated microspheres incorporating virulent reporter bacilli, primary human cells, and an extracellular matrix by using bioelectrospray methodology. Granulomas form within the three-dimensional matrix, and mycobacterial stress genes are upregulated. Pyrazinamide, a vital first-line antibiotic for treating human tuberculosis, kills *M. tuberculosis* in a three-dimensional culture but not in a standard two-dimensional culture or Middlebrook 7H9 broth, demonstrating that antibiotic sensitivity within microspheres reflects conditions in patients. We then performed pharmacokinetic modeling by combining the microsphere system with a microfluidic plate and demonstrated that we can model the effect of dynamic antibiotic concentrations on mycobacterial killing. The microsphere system is highly tractable, permitting variation of cell content, the extracellular matrix, sphere size, the infectious dose, and the surrounding medium with the potential to address a wide array of human infections and the threat of antimicrobial resistance.

## IMPORTANCE

Antimicrobial resistance is a major global threat, and an emerging concept is that infection should be studied in the context of host immune cells. Tuberculosis is a chronic infection that kills over a million people every year and is becoming progressively more resistant to antibiotics. Recent major studies of shorter treatment or new vaccination approaches have not been successful, demonstrating that transformative technologies are required to control tuberculosis. We have developed an entirely new system to study the infection of host cells in a three-dimensional matrix by using bioengineering. We showed that antibiotics that work in patients are effective in this microsphere system but not in standard infection systems. We then combined microspheres with microfluidics to model drug concentration changes in patients and demonstrate the effect of increasing antibiotic concentrations on bacterial survival. This system can be widely applied to address the threat of antimicrobial resistance and develop new treatments.

## INTRODUCTION

The progressive emergence of drug-resistant bacteria poses one of the most pressing threats to human health, with the development of totally resistant bacteria potentially leading to a return to the preantibiotic era (1–3). The pipeline of new antibiotics in development is inadequate to combat the rate of evolution of microbial resistance (4, 5). To develop antibiotics, bacteria have traditionally been studied in broth culture, where bacilli are rapidly dividing under optimal growth conditions. However, an emerging concept is that studying pathogens in the context of the host is vital to fully understanding pathogenesis (6, 7). Interaction with host cells modulates multiple facets of bacterial physiology and causes stress-induced changes in bacterial gene expression (8). In parallel, evidence is accumulating that host cell biology is modulated by three-dimensional (3D) extracellular matrix interactions, regulating key processes in the host-pathogen interaction such as cell survival, phagolysosomal fusion, autophagy, and cytokine secretion (9, 10). In patients being treated for infection, the host-pathogen interaction occurs in three dimensions and antibiotic concentrations vary over time according to drug pharmacokinetics (11). Conversely, the vast majority of *in vitro* studies are done in the absence of human cells, without an extracellular matrix, and at static antibiotic concentrations.

Considering these concepts together, we concluded that a transformative system to address the threat of antimicrobial resistance requires the following elements: primary host cells infected with fully virulent bacteria, culturing within a 3D structure that incorporates a physiological extracellular matrix, and pharmacokinetic modeling of drug concentrations. These criteria represent a significant challenge in the context of virulent organisms because of the high biosafety containment level required and the complexity of bacteria being eluted under flow conditions. We utilized *Mycobacterium tuberculosis*, a pathogen that is inherently resistant to antibiotics and causes tuberculosis (TB) (12), to develop a system that addresses these technical obstacles and have recently reported on an investigation of the host immune response in this system (13).

TB is the leading cause of death from an infectious disease worldwide (14), and over the last 2 decades, multidrug-resistant, extensively drug-resistant, and totally drug-resistant strains have sequentially emerged, posing the specter of a completely untreatable disease (15). Unfortunately, major recent trials of novel treatment-shortening regimens have not been successful (16), indicating that the model systems that were used to inform these approaches do not sufficiently reflect the disease in humans. Furthermore, pyrazinamide (PZA), one of the most critical antibiotics in human TB treatment, would not have been discovered by current screening approaches. Current models are principally reliant on microbiological broth or solid medium culture, 2D culture, zebrafish, and mice (17). Novel PZA-based regimens show promise (18), and so reliably understanding the action of PZA has become critical, principally focused on mutational analysis of PZA resistance (19). These approaches have limitations, especially in the context of PZA’s complex activation, intracellular activity, and uncertain mode of action. For some other drugs, such as cycloserine, nearly all drug susceptibility systems are unreliable. *M. tuberculosis* is an obligate pathogen of humans and has a prolonged interaction with host cells, centered on adaption to survival within an intracellular niche (20). In addition, the host-pathogen interaction is spatially organized (21) and the extracellular matrix influences host cell survival (22), suggesting that a fully humanized system structured in three dimensions with an extracellular matrix is needed to identify novel treatments for TB.

Therefore, we developed a platform utilizing *M. tuberculosis* as the prototype organism. Our system integrated genetically modified virulent reporter bacilli, primary human cells, and a human extracellular matrix by using a bioengineering approach and combined this with a multiparameter longitudinal readout. Within this microsphere system, we demonstrate cellular aggregation and upregulation of mycobacterial stress genes. Critically, PZA is efficacious in the 3D microsphere system but not in standard broth or 2D culture. We then combined microspheres with a microfluidic system to permit pharmacokinetic modeling. We observed more rapid *M. tuberculosis* killing with higher peak antibiotic concentrations, similar to outcomes in patients with TB (23). Therefore, this system models conditions in patients and can be readily applied to a range of drug-resistant organisms to address the global challenge of antimicrobial resistance.

## RESULTS

### Granulomas develop within microspheres, and *M. tuberculosis* stress genes are upregulated.

**FIG 1:**
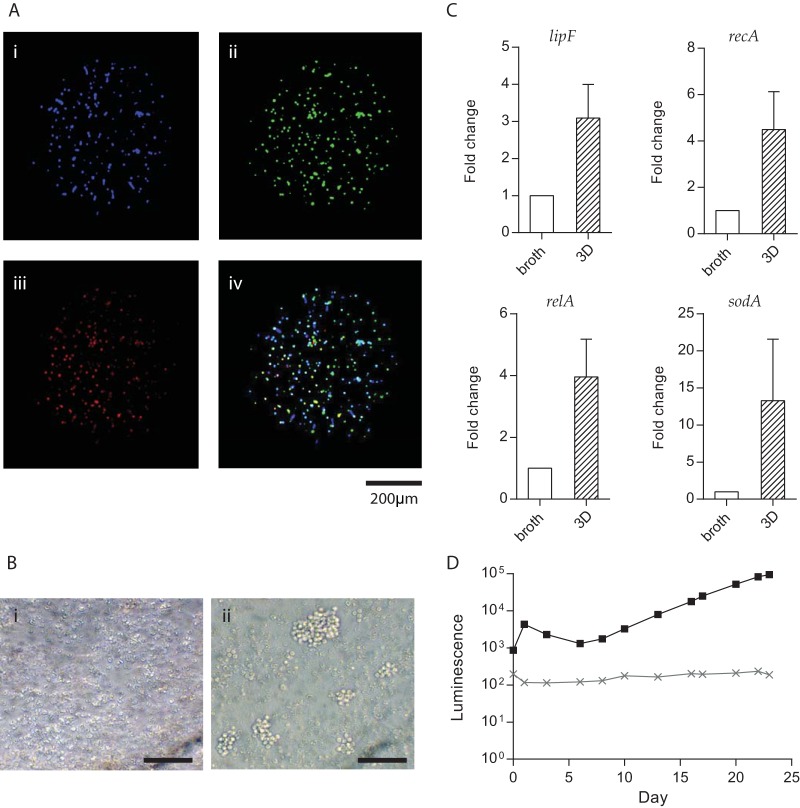
Granulomas form within microspheres, and *M. tuberculosis* stress genes are upregulated. (A) Cellular distribution within microspheres. Primary human PBMCs were separated and fluorescently stained (monocytes blue [i], T cells green [ii]), recombined, and infected with mCherry-expressing *M. tuberculosis* (red [iii]). The overlay (iv) shows early granuloma development at day 4. (B) Large multicellular granulomas form at day 14 in infected microspheres (ii), which are not observed in uninfected microspheres (i), imaged by inverted microscopy. Scale bar, 50 μm. (C) *M. tuberculosis* stress genes are upregulated in the microsphere model compared to 7H9 broth culture. The expression of four stress-related mycobacterial genes was analyzed by RT-qPCR in microspheres at day 14 compared to exponentially growing *M. tuberculosis* (OD_600_ = 0.25) in 7H9 broth. The ΔΔ*C*_*T*_ method was used for relative quantification. Data are presented as fold changes normalized to the *sigA* gene. Data represent the mean results of three independent experiments ± the standard error of the mean. (D) *M. tuberculosis* growth in microspheres monitored by bacterial luminescence, demonstrating the typical *M. tuberculosis* luminescence kinetics of infected PBMCs within microspheres (black). Uninfected PBMCs in the microspheres do not luminesce (gray).

We incorporated primary human cells, virulent *M. tuberculosis*, and type I collagen into 3D microspheres by bioelectrospray methodology (see Movie S1 in the supplemental material). Fluorescent staining of monocytes and T cells, followed by infection with mCherry-expressing *M. tuberculosis*, showed the distribution of cells and bacteria through the microspheres and early granuloma formation from day 4 (Fig. 1A). After 14 days of infection, large cellular aggregates resembling human granulomas developed, while no aggregates formed in uninfected microspheres (Fig. 1B). Granuloma formation was associated with evidence of mycobacterial stress. Multiple stress-related genes were upregulated at day 14 in comparison with *M. tuberculosis* in 7H9 broth culture analyzed by reverse transcription-quantitative PCR (RT-qPCR) (Fig. 1C), including *lipF*, the acid stress real-time response gene; *recA* encoding recombinase A, the key mediator of the SOS response to DNA damage; *relA*, the nutrient stress-related gene; and *sodA*, the oxidative-stress response gene. By infecting cells with genetically modified luminescent *M. tuberculosis* expressing the Lux operon (24), bacterial growth could be monitored longitudinally over time within microspheres in a nondestructive manner (Fig. 1D).

10.1128/mBio.02073-16.7 MOVIE S1  Generation of microspheres. Alginate was stained with bromophenol blue to permit visualization during the bioelectrospray process, which is shown in real time. Download MOVIE S1, AVI file, 2.5 MB. Copyright © 2017 Bielecka et al. 2017 Bielecka et al. This content is distributed under the terms of the Creative Commons Attribution 4.0 International license.

**FIG 2:**
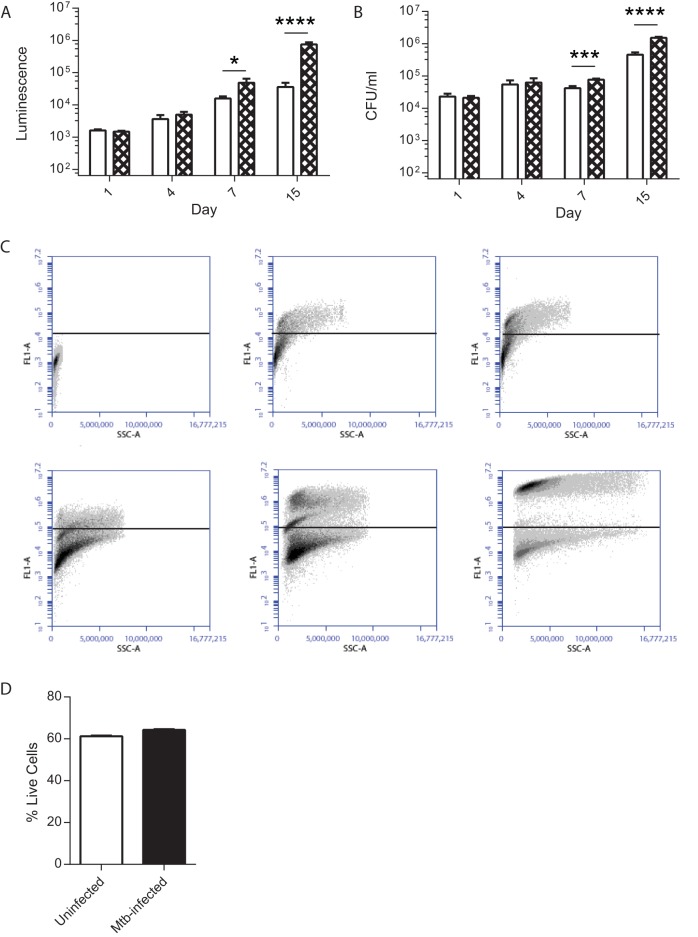
*M. tuberculosis* proliferation within microspheres is intracellular (A, B). PBMCs were infected with luminescent *M. tuberculosis* and incorporated into microspheres. Cells were released by decapsulation, and extracellular and cell-associated bacteria were separated by differential centrifugation. Open bars; extracellular mycobacteria; checkered bars; cell-associated mycobacteria. Mycobacterial location determined by luminescence and colony counting on 7H11 agar demonstrated that bacterial proliferation was principally cell associated (C). PBMCs were infected with GFP-expressing *M. tuberculosis* and incorporated into microspheres. Microspheres were decapsulated, and *M. tuberculosis* localization was analyzed by flow cytometry (i) Uninfected cells. GFP-expressing *M. tuberculosis* cells at time zero (ii), day 1 (iii), day 4 (iv), day 7 (v), and day 15 (vi) show progressive intracellular proliferation. Data are from a representative experiment performed on two occasions in triplicate (D). *M. tuberculosis* infection does not reduce cell viability within microspheres. Cellular survival was measured by the CellTiter-Glo 3D Cell Viability Assay. Data are the mean ± the standard error of the mean of an experiment performed in triplicate on two occasions. *, *P* < 0.05; ***, *P* < 0.001; ****, *P* < 0.0001.

To determine the localization of mycobacteria, we studied microspheres longitudinally. We compared extracellular with cell-associated bacteria by decapsulating microspheres and performing differential centrifugation to separate extracellular mycobacteria from intracellular and cell-adherent mycobacteria. The proportion of cell-associated mycobacteria analyzed by luminescence or CFU counting progressively increased over time (Fig. 2A and B), leading to a 20.7-fold increase in luminescence of cell-associated *M. tuberculosis* relative to extracellular *M. tuberculosis* within microspheres at day 15. Gentamicin treatment in a single experiment demonstrated that cell-associated mycobacteria were almost all intracellular, with no significant reduction in CFU counts after the killing of extracellular bacteria. Similarly, the fluorescence of cells infected with green fluorescent protein (GFP)-expressing *M. tuberculosis* progressively increased over time (Fig. 2C; Fig. S1), demonstrating intracellular proliferation. *M. tuberculosis* infection did not increase cellular toxicity when measured by the CytoTox Glo 3D assay, with no significant difference in viability between the two conditions (Fig. 2D).

10.1128/mBio.02073-16.1 FIG S1  Fluorescence-activated cell sorter analysis strategy for GFP-expressing *M. tuberculosis* associated with cells. Microspheres were generated, and on days 0, 1, 4, 7, and 15, they were decapsulated. Cells were stained for CD14, fixed with 2% paraformaldehyde for 1 h, and then analyzed on a BD Accuri C6 flow cytometer. All events in the high forward and side scatter area gates were then analyzed for CD14 and GFP signals. Columns: i, gating area, cells confirmed to be viable in preliminary experiments; ii, FL4 channel, CD14 antibody; iii, FL1 channel GFP plotted against side scatter; iv, FL1 GFP channel histogram. (A) Unstained, uninfected cells. (B) Uninfected cells stained with an isotype antibody for CD14 antibody, showing no increase in the FL4 channel. (C) Uninfected cells stained with anti-CD14 antibody showing an increase in the FL4 channel. There was only a background signal in the FL1 channel, which detects GFP fluorescence. (D) PBMCs infected with GFP^+^ *M. tuberculosis* stained with isotype control antibody. Infection increases the FL1 signal (iii, iv). (E) PBMCs infected with GFP^+^ *M. tuberculosis* stained with CD14 antibody. A relative decrease in CD14 expression relative to that in uninfected cells at day 1 is observable (ii). *M. tuberculosis* infection increases the FL1 signal. (F) Uninfected PBMCs stained for CD14 at day 15. CD14 expression is reduced (ii). (G) Infected PBMCs at day 15 show an increased CD14 signal (ii) and also a progressive increase in the number of GFP-positive *M. tuberculosis* bacteria associated with cells (iii, iv). Download FIG S1, EPS file, 2.2 MB. Copyright © 2017 Bielecka et al. 2017 Bielecka et al. This content is distributed under the terms of the Creative Commons Attribution 4.0 International license.

### Standard antibiotics kill *M. tuberculosis* under all conditions.

**FIG 3:**
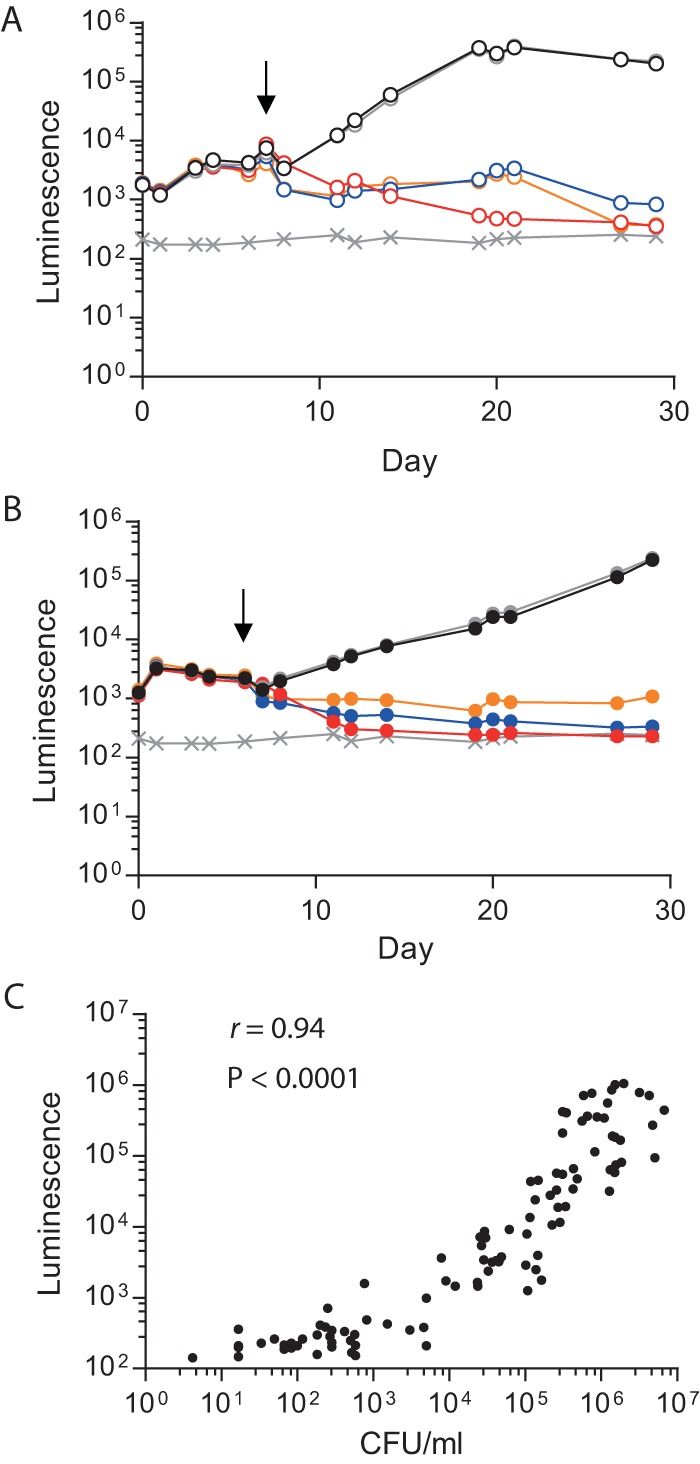
Effects of standard antituberculosis antibiotics on *M. tuberculosis* growth. Antibiotics (rifampin [red, 1 µg/ml], INH [blue, 0.25 µg/ml], and ethambutol [orange, 4 µg/ml]) were added at day 6 to 2D PBMC cultures or the microsphere system, and *M. tuberculosis* growth was monitored by measuring luminescence. *M. tuberculosis* growth was inhibited by all of the antibiotics in both 2D cell cultures (A) and the 3D model (B). *M. tuberculosis* growth was unaffected in the control sample (black) or by the addition of DMSO (gray), which was used as a solvent for rifampin. Symbols: ×, background level of luminescence; black arrows, antibiotic addition. Data are the mean ± the standard error of the mean of an experiment performed in triplicate and are representative of three separate experiments. (C) *M. tuberculosis* luminescence closely correlates with CFU counts on Middlebrook 7H11 agar. Spearman *r* value = 0.94; *P* < 0.0001.

Having demonstrated granuloma formation and the *M. tuberculosis* stress response within microspheres, we first studied standard first-line antibiotics in 2D cell culture and the 3D model to determine the tractability of the model and whether killing efficacy is the same under both conditions. Rifampin, isoniazid (INH), and ethambutol were added to cell culture medium around spheres at physiological concentrations (1, 0.25, and 4 µg/ml, respectively). All three antibiotics inhibited *M. tuberculosis* growth in both 2D and 3D cell culture systems (Fig. 3A and B, antibiotics added at day 6; Fig. S2, antibiotics added at day 1). Rifampin was the most efficacious at killing *M. tuberculosis*, and INH was consistently more efficient at controlling *M. tuberculosis* in the 3D microsphere system than in 2D cell cultures. *M. tuberculosis* growth analyzed by luminescence correlated closely with CFU counts on Middlebrook 7H11 agar (Fig. 3C).

10.1128/mBio.02073-16.2 FIG S2  Standard antibiotics added on day 1 kill *M. tuberculosis* in 2D and 3D systems. Rifampin (red), INH (blue), and ethambutol (orange) were added at 1, 0.25, and 4 µg/ml, respectively, at day 1 to either 2D cell cultures (A) or the 3D microsphere system (B). *M. tuberculosis* growth was inhibited by all of the antibiotics. *M. tuberculosis* growth was unaffected in the control sample (black) or by DMSO (gray). The × line indicates the background level of luminescence. The black arrow indicates the point of antibiotic administration. Data are the mean ± the standard error of the mean of an experiment performed in triplicate and are representative of three separate experiments. Download FIG S2, EPS file, 1.1 MB. Copyright © 2017 Bielecka et al. 2017 Bielecka et al. This content is distributed under the terms of the Creative Commons Attribution 4.0 International license.

### PZA is only efficacious in microspheres and not in broth or 2D cell cultures.

**FIG 4:**
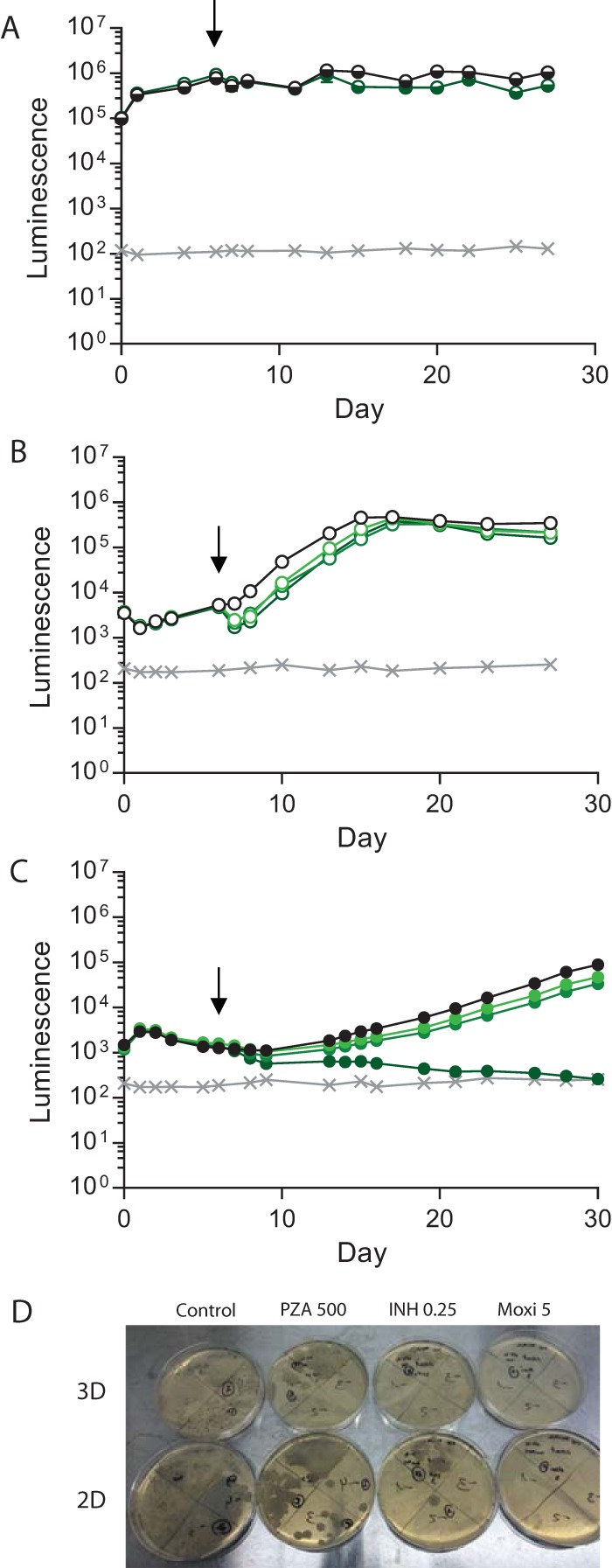
PZA kills *M. tuberculosis* in the 3D model but not in 7H9 broth or 2D cultures. (A) PZA has no effect on *M. tuberculosis* growth in 7H9 broth (dark green, 500 µg/ml) compared with an untreated control (black). (B) PZA has a brief effect on *M. tuberculosis* growth in 2D PBMC cell cultures at 60 µg/ml (light green), 100 µg/ml (medium green), or 500 µg/ml (dark green) in comparison with an untreated control (black), but *M. tuberculosis* growth rapidly recovers. (C) PZA kills *M. tuberculosis* in the 3D system at 500 µg/ml (dark green). Minimal killing of *M. tuberculosis* was observed when 60 µg/ml (light green) or 100 µg/ml (medium green) PZA was added, relative to the control sample (black). Symbols: ×, background level of luminescence; black arrows, antibiotic addition. Data are the mean ± the standard error of the mean of an experiment performed in triplicate and are representative of three separate experiments. (D) Colony counting on 7H11 agar confirms *M. tuberculosis* killing by PZA. Dilutions of control and 2D PZA plates start from a 1:10 dilution, while all other plates start without dilution. Representative plates are shown.

Next, we investigated PZA, which is a key antibiotic in treating human disease but has a poorly defined mechanism of action at the concentration described in epithelial cell lining fluid (25). PZA had no effect on *M. tuberculosis* growth in 7H9 broth without cells at neutral pH (Fig. 4A). In 2D primary cell cultures, PZA had a temporary effect but *M. tuberculosis* growth rapidly recovered (Fig. 4B). Critically, PZA killed *M. tuberculosis* in the 3D microsphere system, with luminescence falling to background levels by day 30 when we used the same antibiotic preparation that had no effect in broth and a transient effect in 2D cultures (Fig. 4C). A similar pattern of efficacy was observed when PZA was added to cultures on day 1, with PZA having no effect in 7H9 broth and a temporary effect in 2D cultures but complete control of *M. tuberculosis* growth in microspheres (Fig. S3). Colony counting on 7H11 agar confirmed that the efficacy of *M. tuberculosis* killing by PZA was equivalent to that of INH and moxifloxacin (Fig. 4D; Fig. S2D).

10.1128/mBio.02073-16.3 FIG S3  PZA kills *M. tuberculosis* more effectively in 3D microspheres than in 2D cultures when added on day 1. (A) PZA at 500 µg/ml (dark green) has no effect on *M. tuberculosis* growth in 7H9 broth in comparison with that in an untreated control. (B) PZA at a concentration of 60 µg/ml (light green), 100 µg/ml (medium green), or 500 µg/ml (dark green) initially significantly inhibited *M. tuberculosis* growth in 2D cell cultures compared to that in untreated controls. However, bacterial regrowth occurred, with *M. tuberculosis* luminescence returning to control levels by day 28. (C) PZA is bactericidal in the 3D system. *M. tuberculosis* growth was inhibited slightly by a low dose of PZA, while 500 µg/ml (dark green) was bactericidal. Gray lines with × symbols indicate the background levels of luminescence. Black arrows indicate points of antibiotic addition. Data are the mean ± the standard error of the mean of an experiment performed in triplicate and are representative of three separate experiments. (D) Bacteria were plated on 7H11 agar on day 28. CFU counts confirm that the luminescence data reflect bacterial loads. Note that dilutions of control and 2D PZA plates start from a 1:10 dilution, while other plates start without dilution. Representative plates are shown. Download FIG S3, EPS file, 1.6 MB. Copyright © 2017 Bielecka et al. 2017 Bielecka et al. This content is distributed under the terms of the Creative Commons Attribution 4.0 International license.

### Second-line antibiotics are most efficacious in 3D microspheres.

**FIG 5:**
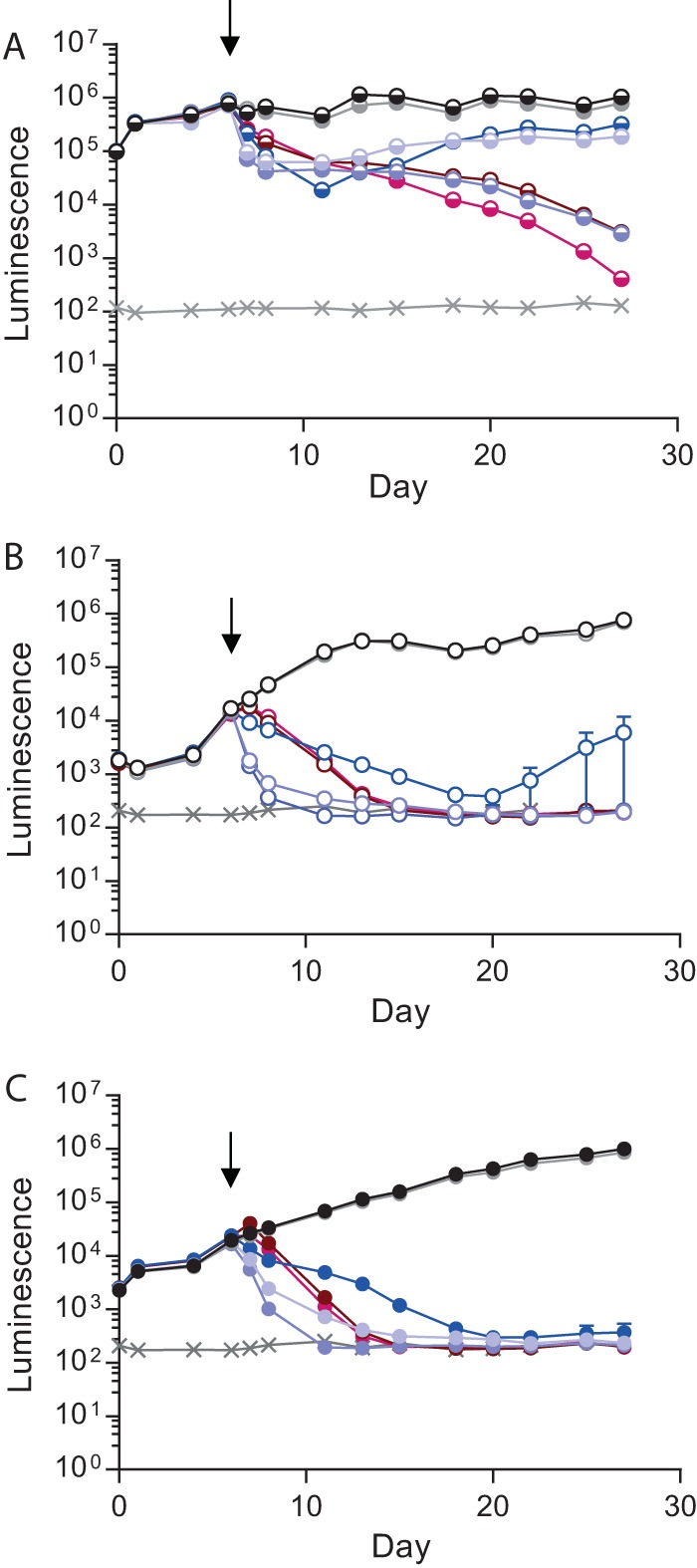
d-Cycloserine has similar effects on *M. tuberculosis* growth in 2D and 3D cultures. (A) *M. tuberculosis* in 7H9 broth. d-Cycloserine at a low concentration (20 µg/ml) had a temporary effect on *M. tuberculosis* growth (light purple) similar to that of INH at 0.25 µg/ml (blue). d-Cycloserine at 200 µg/ml killed *M. tuberculosis* more rapidly than (dark purple) and as effectively as moxifloxacin (brown, 5 µg/ml). Linezolid was the most effective second-line antibiotic (magenta, 24 µg/ml). The diluent DMSO (gray) did not affect *M. tuberculosis* growth relative to that in 7H9 broth only (black). (B) *M. tuberculosis* growth in 2D PBMC cultures. d-Cycloserine at both concentrations inhibited *M. tuberculosis* growth (purple) more rapidly than the other antibiotics (moxifloxacin [brown, 5 µg/ml], linezolid [magenta, 24 µg/ml], and INH [blue, 0.25 µg/ml]). Shown is *M. tuberculosis* growth in control samples (black) and with DMSO (gray). (C) *M. tuberculosis* growth in a 3D cell culture model. d-Cycloserine, linezolid, and moxifloxacin have an efficacy similar to that in a 2D cell culture (purple), while INH (blue) is more consistently bactericidal. Gray lines indicate background levels of luminescence. Black arrows indicate the day antibiotics were added. Data are the mean ± the standard error of the mean of an experiment performed in triplicate and are representative of three separate experiments.

We then examined the effect of the second-line antibiotics d-cycloserine, moxifloxacin, and linezolid, which are of increasing importance with the emergence of drug-resistant TB. In 7H9 broth, d-cycloserine at a low concentration had a minor inhibitory effect but at a high concentration it was as effective as moxifloxacin (Fig. 5A). In the 2D and 3D systems, d-cycloserine effectively killed *M. tuberculosis* at low and high concentrations (Fig. 5B and C). Linezolid and moxifloxacin effectively suppressed *M. tuberculosis* growth under all three conditions. INH, which was included as a control first**-**line antibiotic, consistently killed *M. tuberculosis* in 3D microspheres, but bacterial growth resumed in 7H9 broth and the 2D system. A similar pattern was observed when antibiotics were added at day 1, although the inhibition of *M. tuberculosis* growth by d-cycloserine was more rapid in microspheres than in 2D cultures (Fig. S4). To ensure that the antibiotics were not having a cytotoxic effect on host cells, we analyzed their viability and found no evidence of cytotoxicity after 21 days of culture compared to medium and dimethyl sulfoxide (DMSO) controls (Fig. S5).

10.1128/mBio.02073-16.4 FIG S4  Second-line antibiotics kill *M. tuberculosis* more rapidly in 3D microspheres than in 2D cell cultures when added on day 1. (A) *M. tuberculosis* growth in 7H9 broth. d-Cycloserine at low (20 µg/ml) and high (200 µg/ml) concentrations (light and dark purple, respectively) inhibit *M. tuberculosis* growth. However, bacterial regrowth occurred with the lower dose. INH (blue, 0.25 µg/ml) had a moderate effect on *M. tuberculosis* growth, and regrowth occurred. Moxifloxacin (brown, 5 µg/ml) and linezolid (magenta, 24 µg/ml) were equally effective but with delayed killing in comparison with high-dose d-cycloserine. *M. tuberculosis* growth was unaffected in control samples (black; gray with the diluent DMSO). (B) *M. tuberculosis* growth in 2D cell cultures. d-Cycloserine at a higher dose (dark purple) killed *M. tuberculosis* with an efficacy equal to that of the other antibiotics tested (5 µg/ml moxifloxacin [brown], 24 µg/ml linezolid [magenta], and 0.25 µg/ml INH [blue]). d-Cycloserine at a lower concentration inhibited *M. tuberculosis* growth but with a delay in comparison with the other antibiotics. (C) d-Cycloserine kills *M. tuberculosis* more rapidly in 3D cell cultures. All of the antibiotics investigated were similarly effective against *M. tuberculosis*, with rapid killing and no bacterial regrowth. Gray lines with × symbols indicate background luminescence. Black arrows indicate points of antibiotic addition. Data are the mean ± the standard error of the mean of an experiment performed in triplicate and are representative of three separate experiments. Download FIG S4, EPS file, 1.5 MB. Copyright © 2017 Bielecka et al. 2017 Bielecka et al. This content is distributed under the terms of the Creative Commons Attribution 4.0 International license.

10.1128/mBio.02073-16.5 FIG S5  Antibiotics do not cause cytotoxicity within microspheres. Cellular cytotoxicity was investigated at day 21 within the 3D system with the CellTiter-Glo 3D cell viability assay. No antibiotic significantly changed cellular survival either in the absence of infection (A) or after *M. tuberculosis* infection (B). Similarly, cytotoxicity, analyzed by LDH release, is not different in infected cells treated with antibiotics (C). Download FIG S5, EPS file, 1.2 MB. Copyright © 2017 Bielecka et al. 2017 Bielecka et al. This content is distributed under the terms of the Creative Commons Attribution 4.0 International license.

### Pharmacokinetic modeling by integration with microfluidics.

**FIG 6:**
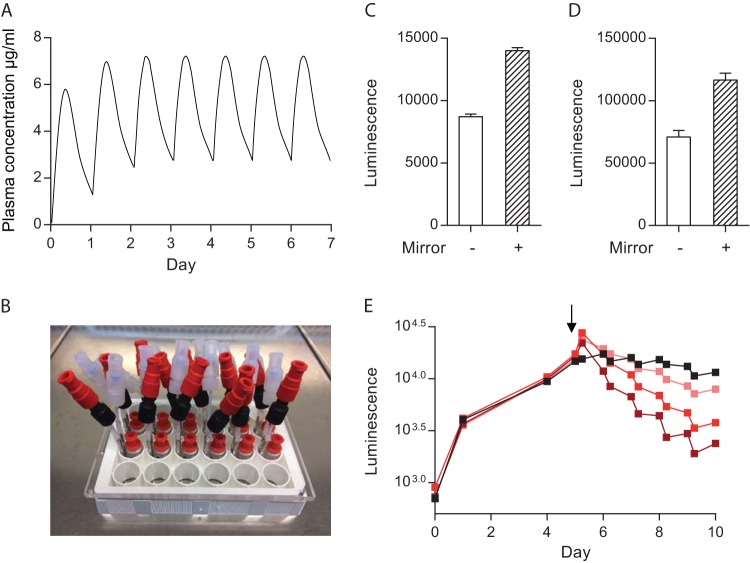
Modeling of antibiotic pharmacokinetics by integrating microspheres with a microfluidic system. (A) Representation of antibiotic pharmacokinetics in human plasma after daily oral administration during treatment. (B) Microfluidic system with two input channels and one exit channel for a 24-well tissue culture plate. (C, D) Placement of a basal mirror doubles the detection of *M. tuberculosis* luminescence by the GloMax Discover plate reader. Luminescence from infected PBMCs in microspheres in a single well in the absence (no fill) or presence (stripes) of a basal mirror for 24-well (C) and 96-well (D) tissue culture plates. (E) Modeling of antibiotic concentration profiles with a microfluidic system. From day 5 (black arrow), various peak concentrations of antibiotics were introduced for 6 h via the fluidic system and then washed out to approximate pharmacokinetics *in vivo*. Increasing rifampin concentrations (0.25 µg/ml [salmon], 1 µg/ml [bright red], and 4 µg/ml [dark red]) progressively accelerated *M. tuberculosis* killing. The black line represents a control sample to which the carrier DMSO was added and identical washes were performed. Three independent experiments were carried out, and the results of a representative experiment are shown.

In patients, antibiotic concentrations fluctuate over time, as opposed to the static concentrations usually studied in the laboratory. Therefore, we integrated the microsphere system with a microfluidic platform to permit modulation of antibiotic concentrations over time to mimic *in vivo* pharmacokinetics in patients during treatment (Fig. 6A). We studied rifampin, as its concentrations in plasma correlate with treatment outcomes (23). We manufactured a microfluidic plate from milled poly(methyl methacrylate) (PMMA), providing each well with two inlets and one outlet, permitting a smooth flow of medium through the wells containing microspheres (Fig. 6B). Initially, bacterial luminescence from the 24-well plate was undetectable on a GloMax DISCover plate reader. To overcome this, we used phenol red-free medium, optimized the microsphere density within wells, and placed a custom-made mirror under the plate. These modifications greatly improved the luminescence readout (Fig. 6C and D), and we were able to monitor bacterial growth from experimental time zero.

To model pharmacokinetics in patients, rifampin at a range of concentrations (0.25, 1, and 4 µg/ml) was used to irrigated wells from day 5, incubated for 6 h, and then washed out to leave a minimal antibiotic concentration overnight. A stepwise increase in the rifampin concentration in individual wells produced dose-dependent killing of *M. tuberculosis* (Fig. 6E). The highest 6-h peak rifampin concentration caused *M. tuberculosis* killing equal to that of a constant 1 µg/ml antibiotic concentration or that of irrigation with medium with rifampin at 1 μg/ml (Fig. S6A). Luminescence increased overnight in the absence of antibiotics, resulting in a sawtooth pattern of killing that did not occur at a fixed antibiotic concentration and demonstrated rapid recovery of mycobacterial growth once antibiotic pressure was removed. Colony counts on 7H11 agar confirmed that the luminescence data reflected the total bacterial load (Fig. S6B).

10.1128/mBio.02073-16.6 FIG S6  High-dose rifampin for 6 h/day has efficacy equal to that of a constant standard antibiotic concentration. (A) *M. tuberculosis* growth inhibition in the microfluidic system at different concentrations of rifampin (0.25 [salmon], 1 [bright red], and 4 [dark red] µg/ml). A constant 1 µg/ml rifampin (blue) or addition of rifampin at a concentration of 1 µg/ml without daily washes (checkered box) produced inhibition of *M. tuberculosis* growth equivalent to that of a 4 µg/ml temporary peak concentration of rifampin, but no overnight regrowth occurred. Controls with DMSO (black) and without DMSO (white squares) were included. (B) Colony counts on 7H11 agar confirm that the luminescence data reflect the total *M. tuberculosis* loads within microspheres. Download FIG S6, EPS file, 2 MB. Copyright © 2017 Bielecka et al. 2017 Bielecka et al. This content is distributed under the terms of the Creative Commons Attribution 4.0 International license.

## DISCUSSION

Antimicrobial resistance is rapidly emerging as one of the most pressing challenges to global society (2, 3). Innovative approaches to studying the host-pathogen interaction are urgently needed to identify novel treatment approaches that counter the evolution of drug-resistant bacteria. To achieve this, an emerging paradigm is that bacteria should be studied in the context of the host (6). We hypothesized that a transformative platform will require multiple elements that are not currently available in a single system: virulent reporter bacteria, primary human cells, an extracellular matrix, 3D organization of the host-pathogen interaction, and pharmacokinetic modeling. We combined diverse methodologies, including genetically modified bacteria, primary human cell culture, electrostatic microsphere generation, multiparameter readouts, and microfluidics, to develop a platform with all of the requisite elements that could be used in a biosafety level 3 containment environment. In our investigation of the host immune response, we showed that host cell survival is improved in the 3D microsphere model, cellular aggregates form, and host-directed therapies can be studied (13). Here, we investigated the system from the pathogen’s perspective and demonstrated that mycobacterial stress genes are upregulated and key antibiotics used to treat human disease are more efficacious in the microsphere system than in standard culture. The nondestructive readouts permit longitudinal analysis over prolonged periods and real-time pharmacokinetic modeling. This platform has the potential to revolutionize antibiotic discovery and to replace suboptimal animal model systems based on inappropriate host-pathogen combinations.

Miniaturized organoid systems and lab-on-a-chip technologies are rapidly evolving fields for drug discovery, resulting from the widespread belief that studying individual cells or infectious organisms in isolation does not sufficiently reflect conditions *in vivo* (26, 27). The bioelectrosprayer can produce large numbers of identical microspheres rapidly in a patient-relevant model, fulfilling a principal requirement of such a system (28). 2D systems with flow have been developed to study respiratory epithelial cell infection (29), and while several TB cellular systems have been reported (30–34), none combine 3D culture, collagen, and the potential for high-throughput or longitudinal analysis. The microsphere system is highly tractable, with the ability to modulate infectious organism and doses, host cellular content, the extracellular matrix, microsphere size, and the surrounding medium dynamically.

The need for new antibiotics for TB is particularly acute, given the progressive emergence of drug resistance, recent disappointments in treatment-shortening regimens, and the ongoing global toll of infection (14). PZA is one of the most important antibiotics used to treat human disease, but it was discovered serendipitously in the 1950s and indeed would have not been found by current screening approaches based on the sequential study of MICs in broth culture, murine infections, and human disease (17). PZA has a complex mechanism of action, requiring intracellular acidification. We demonstrated that *M. tuberculosis* stress genes were upregulated in microspheres and that PZA killed *M. tuberculosis* in microspheres at neutral pH but not in standard 2D culture systems at a concentration found in epithelial lining fluid (25). This confirms that, within microspheres, bacilli are in a PZA-sensitive compartment and demonstrates the potential to identify other compounds only active against stressed mycobacteria in the correct microenvironment. Similarly, we found that INH was consistently more effective in the 3D system that in the 2D system, with a more rapid fall in luminescence and no failed treatments, supporting the relevance of the model to human TB. Therefore, the system can identify drug resistance in a more clinically relevant fashion and can be used to study novel regimens with variable concentrations in combination rather than single new agents at static concentrations.

The microsphere system has potential for high-throughput use, as >5,000 microspheres can be generated from a single blood donor within an hour and the diameter is compatible with a 384-well format. Mycobacterial killing curves during antibiotic treatment of patients suggest that there are diverse populations within patients’ lungs (35), and therefore, the system can be used to study the separate physiological conditions that drive these within the same experiment, such as hypoxia and nutritional stress. Encapsulation within microspheres permits integration with a microfluidic system without cells and bacteria being lost during irrigation, resolving a significant technical hurdle for pharmacokinetic studies. We were able to show more rapid killing with increased rifampin concentrations, consistent with findings in patients (23, 36). Microfluidics have been used to develop an array of organ-on-a-chip models (37), but we are not aware of development to study virulent containment level 3 pathogens such as *M. tuberculosis*, which represents additional challenges because of the infection risk. The hollow-fiber model has been used to perform advanced pharmacokinetic modeling, but PZA is only efficacious in this system after acidification to pH 5.8 (38) and so cannot be studied in combination with other agents. Future potential developments for the bioelectrospray platform include dual encapsulation to permit a central lipid-rich caseous core, generating an additional layer of flexibility and modeling drug penetration into necrotic foci (39). Development of the microfluidic plate will permit optimization of combinations of multiple antibiotics in a fully humanized system, with pharmacokinetic modeling of each antibiotic within wells, to identify the best combinations with which to go forward to clinical trials (34).

We developed a bioengineered cell culture platform that replicates key features of human disease and incorporates primary human cells, an extracellular matrix, a 3D structure, virulent bacteria, and pharmacokinetic modeling. The microsphere system is highly tractable, permitting variation of cell content, the extracellular matrix, sphere size, the infectious dose, and the surrounding medium with the potential to address a wide array of human infections. The system can equally be applied to diverse inflammatory and malignant human diseases. Integration with molecular microbiology techniques and clustered regularly interspaced short palindromic repeat gene editing will provide genetically tractable host-pathogen pairings. Therefore, this platform has global applicability to address the threat of antimicrobial resistance and deliver new treatments.

## MATERIALS AND METHODS

### Bacterial strains, culture conditions, and chemicals.

Bioluminescent *M. tuberculosis* H37Rv (*M. tuberculosis* Lux) (24) and mCherry-expressing *M. tuberculosis* H37Rv (40) were cultured in Middlebrook 7H9 medium (BD Biosciences UK, Oxford, United Kingdom) supplemented with 10% ADC Enrichment (Scientific Laboratory Supplies, Nottingham, UK), 0.2% glycerol, 0.02% Tween 80, and kanamycin (25 µg/ml) or hygromycin (50 µg/ml), respectively. For all experiments, cultures were grown to an optical density at 600 nm (OD_600_) of 0.6 (approximately 1 × 10^8^ CFU/ml). Bacterial growth in 7H9 broth was monitored by measuring luminescence (GloMax 20/20 Single Tube luminometer; Promega, United Kingdom). Chemicals were purchased from Sigma-Aldrich unless stated otherwise.

### Human PBMC isolation and infection.

Ethical approval for these studies was provided by the National Research Ethics Service Committee South Central—Southampton A, ref. 13/SC/0043. Peripheral blood mononuclear cells (PBMCs) were isolated from single-donor buffy coats from the National Health Service Blood and Transplant, Southampton, United Kingdom. Leukocytes were isolated by density gradient centrifugation over Ficoll-Paque (GE Healthcare Life Sciences, United Kingdom). Isolated PBMCs were infected with *M. tuberculosis* Lux at a multiplicity of infection (MOI) of 0.1 and kept overnight at 37°C in a 5% CO_2_ incubator in RPMI 1640 medium supplemented with 10 μg/ml ampicillin, 2 mM glutamine, 25 µg/ml kanamycin, and 10% fetal bovine serum (Labtech International Ltd.). The next day, infected PBMCs were transferred from vented flasks to 50-ml Falcon tubes after detachment with Versene solution (Sigma) for 10 min and scraping. After Hanks balanced salt solution (HBSS) without Ca/Mg (Gibco) was added, cells were centrifuged at 320 × *g* for 8 min at 4°C and the supernatant was decanted. The pelleted cells were resuspended in appropriate volumes of RPMI 1640 medium supplemented with 10 μg/ml ampicillin, 2 mM glutamine, 25 µg/ml kanamycin, and 10% human AB serum (Sigma), referred to as complete RPMI medium.

### 2D culture.

Infected cells were resuspended in 50 ml of complete RPMI medium, and 1 ml was equally distributed into 2-ml Eppendorf tubes at a final concentration of 3 × 10^6^/ml. Cultures were incubated at 37°C in 5% CO_2_. *M. tuberculosis* luminescence was monitored with a GloMax 20/20 luminometer. Antibiotics were added at predetermined time points. For colony counts, cultures were treated with 1% saponin in HBSS and bacteria were plated onto 7H11 agar at serial dilutions. For RT-qPCR analysis, infected cells were plated into six-well plates at a final concentration of 2.5 × 10^6^/ml.

### 3D culture.

Infected cells were resuspended in complete RPMI medium, mixed with sterile alginate-collagen at 1 × 10^6^ cells/ml, and injected into an Electrostatic Bead Generator (Nisco, Zurich, Switzerland) to form microspheres via a Harvard syringe driver as described previously (41). After generation, microspheres were equally distributed into 2-ml Eppendorf tubes (microsphere volume, 0.4 ml), immersed in 1 ml of complete RPMI medium, and incubated at 37°C in 5% CO_2_. *M. tuberculosis* luminescence was monitored with a GloMax 20/20 luminometer. For CFU counts, microspheres were dissolved in 55 mM sodium citrate–10 mM EDTA with 1% saponin in HBSS and bacteria were plated onto 7H11 agar. For RT-qPCR analysis, microspheres were cultured in 50-ml Falcon tubes (microsphere volume, 10 ml) in complete RPMI medium.

### Immunofluorescence and confocal imaging.

PBMCs were separated into monocytes and lymphocytes with MACS Cell Separation Columns (Miltenyi Biotec, Inc., Surrey, United Kingdom). Cells were then labeled with CellTracker Blue or CellTrace CFSE (Thermo, Fisher Scientific, United Kingdom) separately in accordance with the manufacturer’s recommendations before infection with mCherry-expressing *M. tuberculosis* H37Rv (40) at an MOI of 0.1. Microspheres were generated and fixed in 4% paraformaldehyde after 4 days. Confocal images were acquired on a Leica TCS SP5 confocal microscope and processed with ImageJ 1.5 0d (NIH, Bethesda, MD).

### Transcription analysis by RT-qPCR.

For bacteria grown in 7H9 broth (OD_600_ of 0.25) and 2D cultures, total RNA was extracted by centrifugation at 13,000 rpm for 10 min and the addition of 500 µl of RNAprotect Bacteria Reagent (Qiagen). The resuspended pellet was left for 10 min at room temperature prior to repeat centrifugation and resuspension of the pellet in 1 ml of TRIzol (Life Technologies, Inc.) and stored at −80°C. For 3D cultures, RNAlater solution (Ambion) was used to preserve RNA overnight at 4°C. Cells were decapsulated with 100 mM sodium citrate and centrifuged at 3,000 × *g* for 30 min, and the pellet was resuspended in 1 ml of TRIzol (Life Technologies, Inc.) and stored at −80°C until use. Thawed samples were transferred to Lysis Matrix B tubes containing 0.1-mm silica beads (Q-Biogene) and homogenized in a MagnaLyser instrument (Roche) at 4,000 rpm for 5 × 45 s with incubation on ice for 1 min between homogenizations. Samples were centrifuged for 1 min at 16,100 × *g* at 4°C, and the supernatant was transferred to a new Eppendorf tube. After phenol-chloroform extraction, the nucleic acids were precipitated with isopropanol, washed with 75% ethanol, air dried for 10 to 15 min, and finally resuspended in nuclease-free water (Fisher Scientific). Genomic DNA was removed with a DNA-free kit (AM1906; Ambion) in accordance with the manufacturer’s instructions. RNA was further purified with the Qiagen RNeasy minikit (Qiagen), subjected with on-column DNase digestion with the RNase-free DNase set (79254; Qiagen), repurified with an RNeasy minikit, and eluted in 50 µl of RNase- and DNase-free water (Fisher Scientific). The first-strand cDNA was synthesized in 10-µl reaction volumes with the High Capacity cDNA RT kit (Applied Biosystems). The cDNA samples were diluted 1:3 in nuclease-free water, and real-time qPCR was performed with 10-µl reaction volumes containing FastStart Universal Probe Master with Rox (Roche), LNA-based probe (designed with the Universal ProbeLibrary System Technology [Roche]) (Table 1), oligonucleotides (Sigma) (Table 1), and 1 µl of a cDNA preparation. Reactions were run on a 7900HT Fast real-time PCR system (Applied Biosystems) with the following program: 2 min at 50°C, 10 min at 95°C, and 40 cycles of 15 s at 95°C and 1 min at 60°C. All samples were amplified in triplicate, and threshold cycle (*C*_*T*_) values of ≥40 were considered negative. Expression data were normalized to the *M. tuberculosis* housekeeping gene *sigA*, and relative quantifications were carried out by the ΔΔ*C*_*T*_ method.

**TABLE 1:** Primers and probes used in this study

Primer or probe	5′–3′ sequence
lipF-FOR	ATGAGCCGCTCGACCATA
lipF-REV	GAGCCGGAAACGTGAATAAG
Roche UPL LNA-probe 160	FAM^a^-TGCCGCCG-dark quencher dye
recA-FOR	AGGAGAATGCCCGCAACT
recA-REV	CTTCTTCTCGATCTCGTCAGC
Roche UPL LNA-probe 22	FAM-TGGTGGAG-dark quencher dye
relA-FOR	CGCATCATCGAGGTGCTAT
relA-REV	CCTGGATTGCCACCAGAA
Roche UPL LNA-probe 152	FAM-TCGCCGTC-dark quencher dye
sodA-FOR	TGGCCGAATACACCTTGC
sodA-REV	GAGATGTGCGGTTCCAGTG
Roche UPL LNA-probe 85	FAM-GACCTGGA-dark quencher dye
sigA-FOR	AGCTGGCCAAAGAGATGGA
sigA-REV	GGGCGTATTGCTGGATTTC
Roche UPL LNA-probe 133	FAM-GGAGAAGG-dark quencher dye

a FAM, 6-carboxyfluorescein.

### Eukaryotic cell viability assay.

Microspheres containing PBMCs alone or *M. tuberculosis*-infected PBMCs were incubated in 96-well plates for 21 days. Cell viability was analyzed at day 21 with the CellTiter-Glo 3D Cell Viability Assay (Promega) in accordance with the manufacturer’s instructions. Luminescence was analyzed by a GloMax Discover 96-well plate reader (Promega, United Kingdom). To measure cell toxicity, lactate dehydrogenase (LDH) release was analyzed by a colorimetric activity assay (Roche, Burgess Hill, United Kingdom).

### Analysis of *M. tuberculosis* location.

PBMCs were infected with luminescent *M. tuberculosis* and incorporated into microspheres. At predefined time points, microspheres were decapsulated and cell-associated *M. tuberculosis* was pelleted by centrifugation at 380 × *g* for 8 min as previously described (42–44). At days 7 and 15, additional samples of cell-associated *M. tuberculosis* were treated with 100 µg/ml gentamicin for 90 min at 37°C in a 5% CO_2_ incubator to remove noninternalized bacteria and then washed with phosphate-buffered saline (PBS). Mycobacterial location was analyzed by measuring luminescence in the supernatant and pellet and also by colony counting on Middlebrook 7H11 agar. For flow cytometry, PBMCs were infected with GFP-expressing *M. tuberculosis* at an MOI of 0.1. Microspheres were made as described above, and on days 0, 1, 4, 7, and 15, microspheres were decapsulated and stained with allophycocyanin-conjugated anti-human CD14 antibody (ImmunoTools, Friesoythe, Germany). Cells were fixed with 2% paraformaldehyde and analyzed on a BD Accuri C6 flow cytometer. All events in the high forward and side scatter areas stained with CD14 were included in the analysis. Flow cytometry data were analyzed with BD Accuri C6 software (ver. 1.0.264.21). Experiments were done at least two times in triplicate.

### Microfluidic system manufacture.

The lid template was based on the original plate lid (Berthold Technologies, United Kingdom). The lid was manufactured from a 5-mm-thick PMMA sheet (Weatherall Equipment & Instruments Ltd.) by micromilling on a ProtoMat 100 micromill (LPKF Laser & Electronics AG, Garbsen, Germany). The tools used for fabrication were a 3.00-mm end mill and a 1.59-mm drill (ACS Industries United Kingdom). The 3.00-mm cutting tool was used to cut out the holding sockets for the Iso-Disc syringe filters (PTFE-4-4, 4 mm [diameter] by 0.45 μm; Supelco, USA) and to cut out the exact 127.90- by 85.85-mm outline of the lid. The inlets for each well were created by drilling pairs of holes through with the 1.59-mm drill and then inserting 30-mm (length) by 0.87-mm (inner diameter) by 1.59-mm (outer diameter) stainless steel tubing (Swagelok, United Kingdom). The stainless steel tubing was terminated with polytetrafluoroethylene tubing (0.75-mm inner diameter) to luer lock syringe connectors. The outlet port was designed to accommodate the Iso-Disc syringe filter. Three 0.15-mm holes were drilled through each Iso-Disc syringe filter to allow withdrawal of the liquid from each well during experiments with a 1-ml syringe (via the outlet port).

### Microfluidic experiments.

For microfluidic experiments, microspheres were placed in 24-well plates (Berthold Technologies, United Kingdom) with RPMI without phenol red (Gibco) supplemented with 10 μg/ml ampicillin, 2 mM glutamine, 25 µg/ml kanamycin, and 10% human AB serum (Sigma). *M. tuberculosis* Lux luminescence was monitored with a GloMax Discover plate reader (Promega, United Kingdom). Rifampin was added to cultures at either day 4 or 5. At 9 a.m. each day, wells were treated with different doses of antibiotic, and after 6 h, wells were irrigated five times with RPMI. A custom-made mirror was placed under the 24-well clear-bottom plate to maximize luminescence collection for detection.

### Statistical analyses.

Statistical analyses were preformed with GraphPad Prism. Differences were considered significant at *P* < 0.05.
